# Supplementary material for: Fragment-based drug nanoaggregation reveals drivers of self-assembly
Source: Nat Commun. 2023 Dec 14;14:8340. doi: 10.1038/s41467-023-43560-0 (PMC10721832; doi:10.1038/s41467-023-43560-0)
Supplement: Supplementary file 1 — Supplementary Information [file 41467_2023_43560_MOESM1_ESM.pdf]

Supplementary Information for:

Fragment-Based Drug Nanoaggregation Reveals Drivers of Self-Assembly

Chen Chen[1,2,3], You Wu[1,2,3], Shih-Ting Wang[4], Naxhije Berisha[1,5,6], Mandana T. Manzari[1], Kristen Vogt[1,2,3], Oleg Gang[4,7,8], and Daniel A. Heller[1,2,3,*]

[1]Molecular Pharmacology Program, Memorial Sloan Kettering Cancer Center, New York, NY 10065, USA
[2]Graduate School of Medical Sciences, Weill Cornell Medicine, New York, NY 10065, USA
[3]Tri-Institutional PhD Program in Chemical Biology, Memorial Sloan Kettering Cancer Center, New York, NY 10065, USA
[4]Center for Functional Nanomaterials, Brookhaven National Laboratory, Upton, NY 11973, USA
[5]The Graduate Center of the City University of New York, NY, 10016, USA
[6]Department of Chemistry, Hunter College, City University of New York, New York, 10065, USA
[7]Department of Chemical Engineering, Columbia University, New York, NY, 10027, USA
[8]Department of Applied Physics and Applied Mathematics, Columbia University, New York, NY, 10027, USA

*Corresponding author: hellerd@mskcc.org

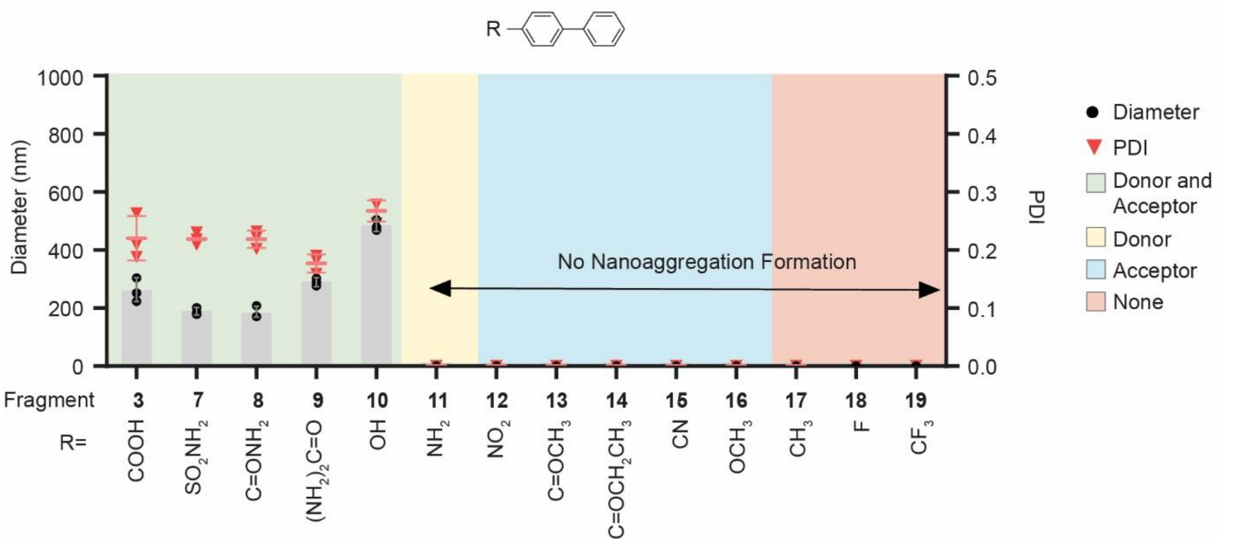

Supplementary Figure 1. Fragment-based drug nanoaggregation assessment with ICG. Average diameter of nanoaggregates formed using biphenyl fragments with various functional group substituents measured by DLS, N=3 biological replicates. Error bar is standard deviation. All bars are presented as mean values with error bars as the standard deviation. Source data are provided as a Source Data file.

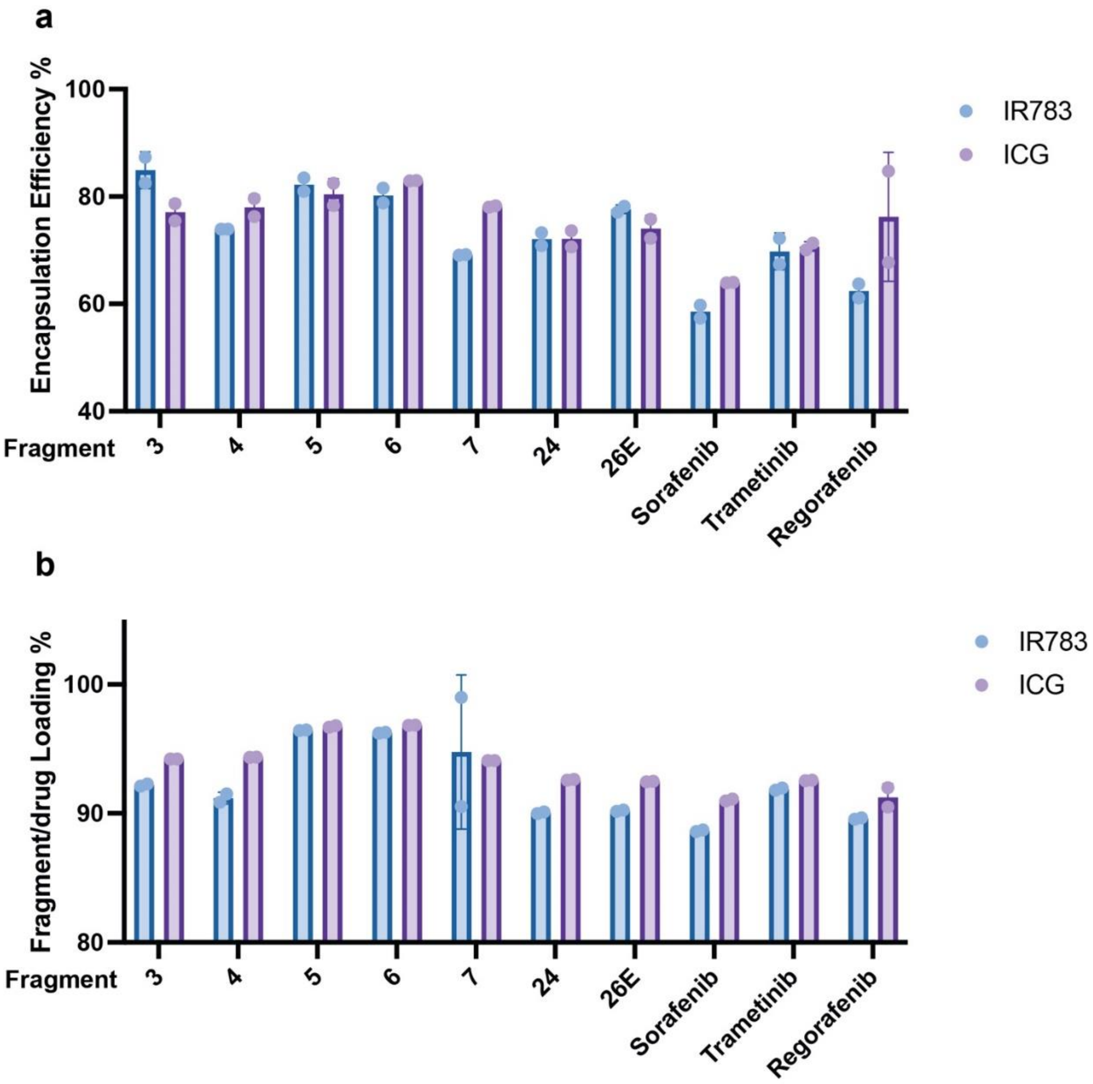

Supplementary Figure 2. Nanoaggregate encapsulation efficiency and fragment/drug loading percent. A selection of fragments and drugs that formed nanoaggregates with IR783 and ICG, characterized for nanoaggregate encapsulation efficiency (a) or fragment/drug loading percent (b). Nanoaggregate encapsulation efficiency (*w*/*w*, %) represents the mass percent of fragments or drugs that ended up in the nanoaggregates; fragment/drug loading percent (*w*/*w*, %) denotes the mass percentage of fragments or drugs in nanoaggregates. N = 2 biological replicates. All bars are presented as mean values with error bars as the standard deviation. Source data are provided as a Source Data file.

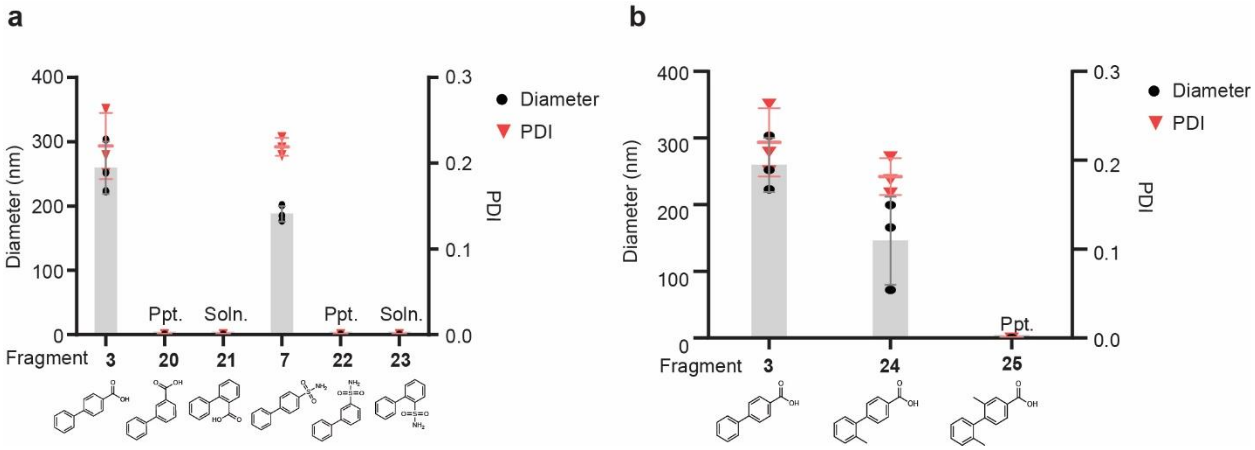

Supplementary Figure 3. Fragment-based drug nanoaggregation assessment with ICG. a-b, The size distribution of regioisomerically substituted biphenyl fragments with ICG in DLS, N=3 biological replicates. Soln. indicates a solution sample with an insufficient count rate in DLS; Ppt. indicates a large precipitate sample with a hydrodynamic diameter larger than 500 nm or PDI more than 0.3 in DLS. Error bar is standard deviation. All bars are presented as mean values with error bars as the standard deviation. Source data are provided as a Source Data file.

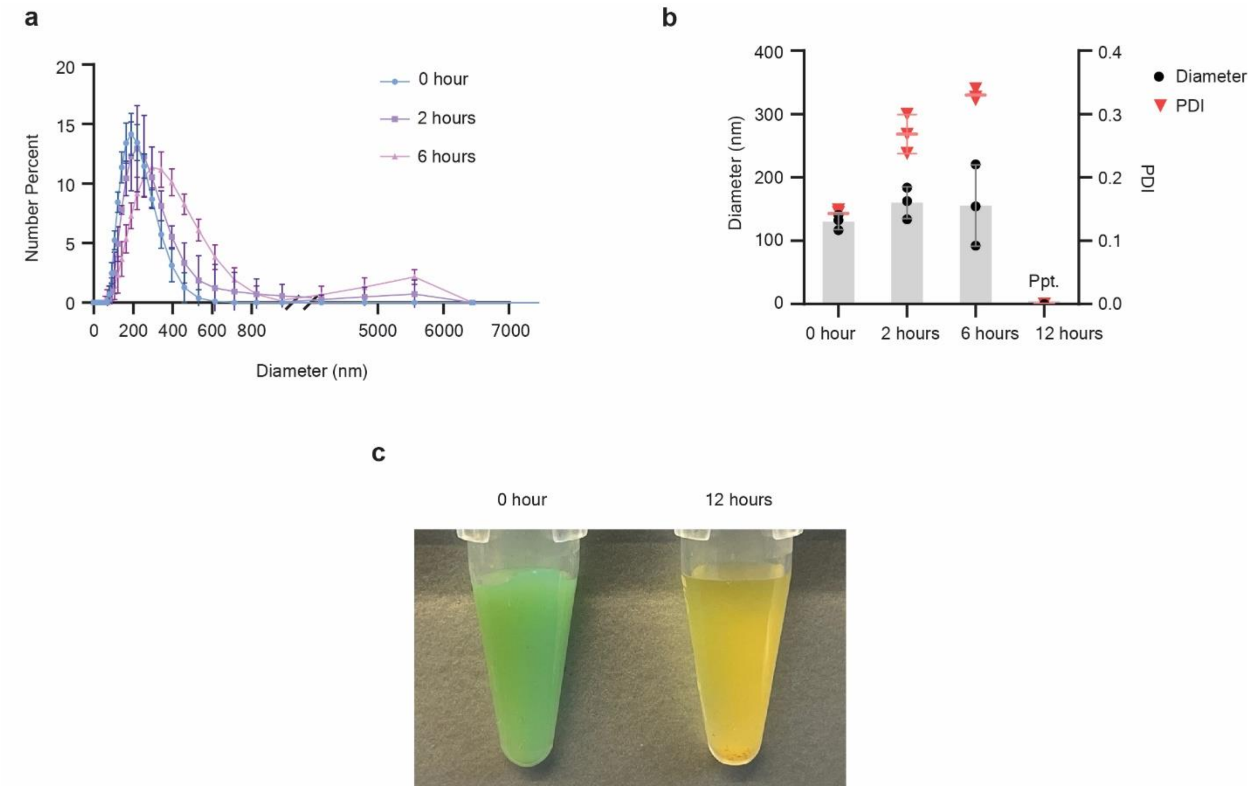

Supplementary Figure 4. Isomerization of 4-phenyldiazenylbenzoic acid nanoaggregates. a, The size distribution of the trans-rich 4-phenyldiazenylbenzoic acid nanoaggregates in DLS with 0, 2, and 6 hours of UV exposures. N=3 biological replicates. b, 4-phenyldiazenylbenzoic acid nanoaggregate formability under 0, 2, 6 and 12 hours of UV exposures (365 nm). N=3 biological replicates. c, 4-phenyldiazenylbenzoic acid nanoaggregates before and after 12 hours exposure to the UV light (365 nm). All bars are presented as mean values with error bars as the standard deviation. Source data are provided as a Source Data file.

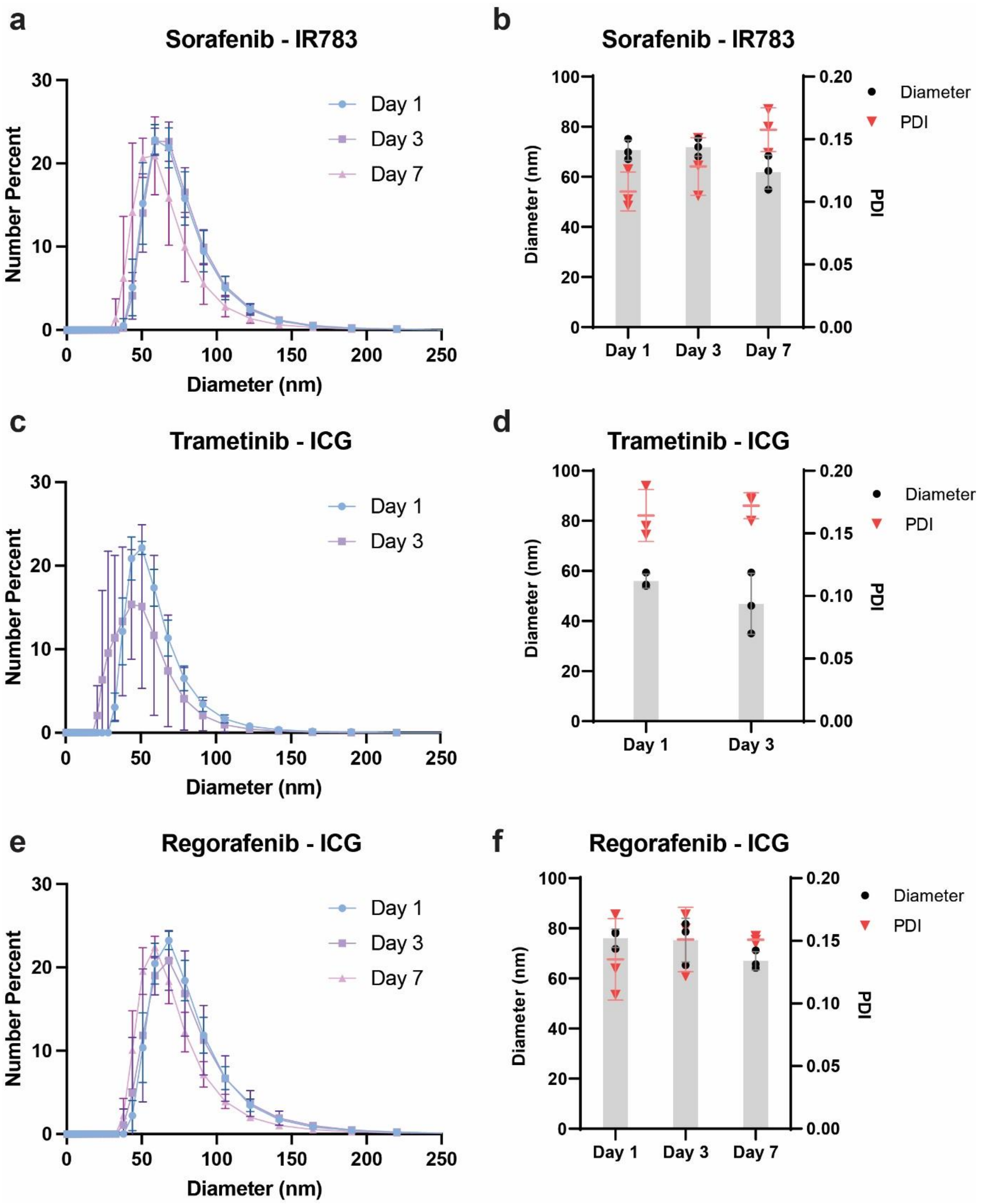

Supplementary Figure 5. Stability of Drug-Encapsulated Nanoaggregates. The size distribution, average diameters and polydispersity index (PDI) of the sorafenib-IR783 (a-b), trametinib-ICG (c-d) and regorafenib-ICG nanoaggregates (e-f) by DLS.. N=3 biological replicates. All bars are presented as mean values with error bars as the standard deviation. Source data are provided as a Source Data file.

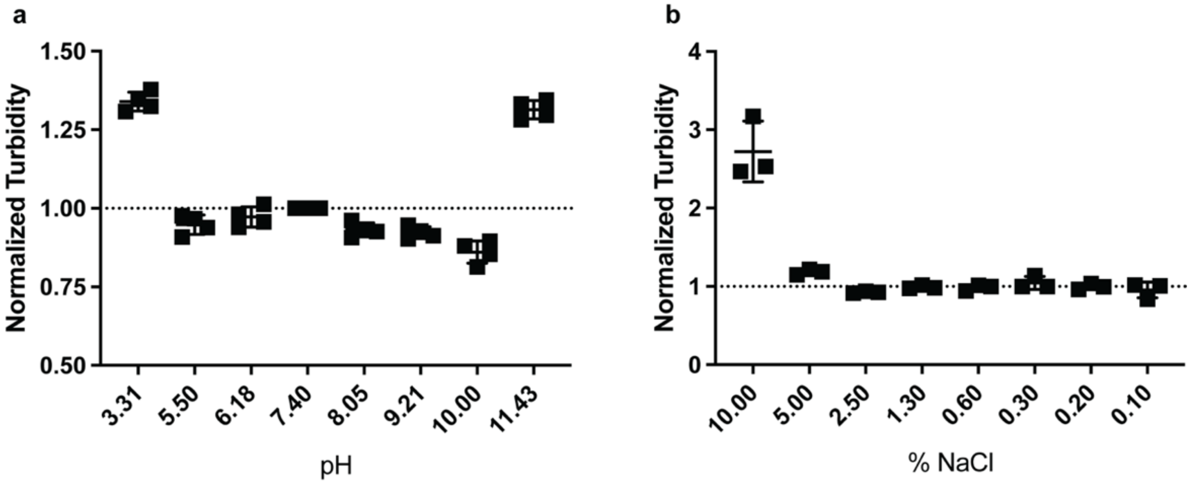

Supplementary Figure 6. Turbidity assessments of sorafenib-IR783 nanoaggregates in (**a**) a range of buffer pH values and (**b**) in a range of salt concentrations in water (weight per volume percentage concentration). Turbidity was normalized to pH=7.4 in panel a and was normalized to water in panel b. N=3 biological replicates. All data are presented as mean values with error bars as the standard deviation. Source data are provided as a Source Data file.

a.

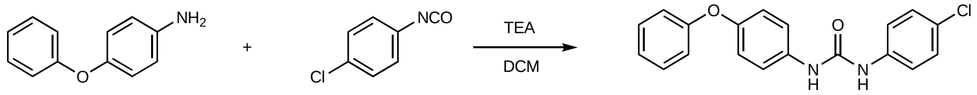

Fragment 29: White solid (73%, 1 step).

^{1}H NMR (500 MHz, DMSO-d_6) δ 8.78 (s, 1H), 8.69 (s, 1H), 7.51 – 7.43 (m, 4H), 7.39 – 7.28 (m, 4H), 7.08 (tt, J = 7.4, 1.1 Hz, 1H), 7.02 – 6.89 (m, 4H).

^{13}C NMR (126 MHz, DMSO-d_6) δ 157.58, 152.48, 150.77, 138.74, 135.47, 129.89, 128.58, 125.25, 122.76, 120.06, 119.72, 119.67, 117.61.

b.

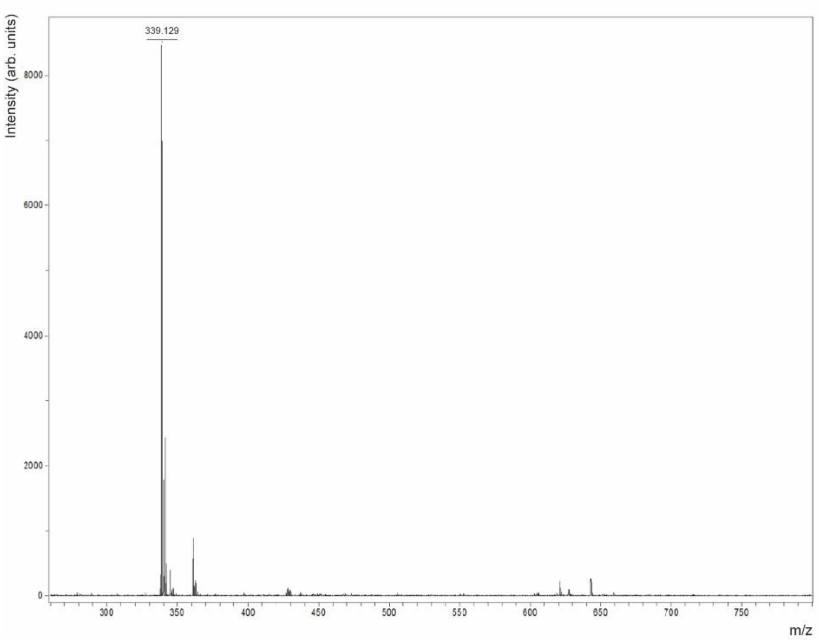

c.

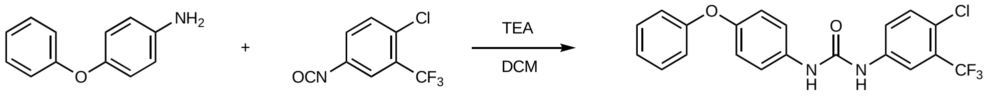

Fragment 30: White solid (61%, 1 step).

^{1}H NMR (500 MHz, DMSO-d_6) δ 9.14 (s, 1H), 8.85 (s, 1H), 8.11 (d, J = 2.4 Hz, 1H), 7.67 – 7.57 (m, 2H), 7.55 – 7.45 (m, 2H), 7.43 – 7.32 (m, 2H), 7.09 (t, J = 7.4 Hz, 1H), 7.04 – 6.89 (m, 4H).

^{13}C NMR (126 MHz, DMSO-d_6) δ 157.53, 152.49, 151.13, 139.42, 135.10, 131.95, 129.91, 123.00, 122.84, 120.48, 119.66, 117.71.

d.

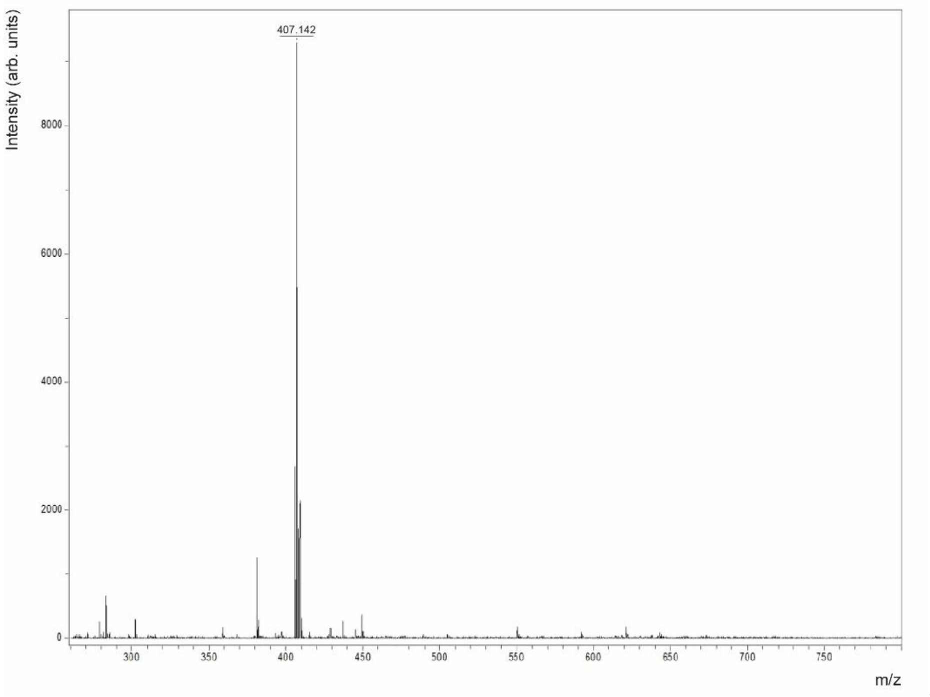

Supplementary Figure 7. The preparation and mass spectrometry of (a-b) fragment **29** and (c-d) fragment **30**.

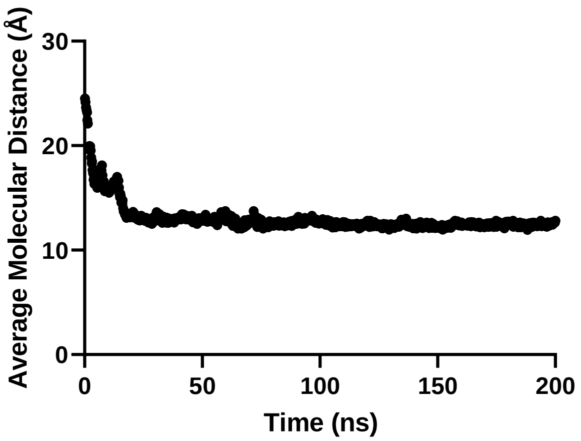

Supplementary Figure 8. Average molecular distance calculated at each frame of the MD simulation. Source data are provided as a Source Data file.

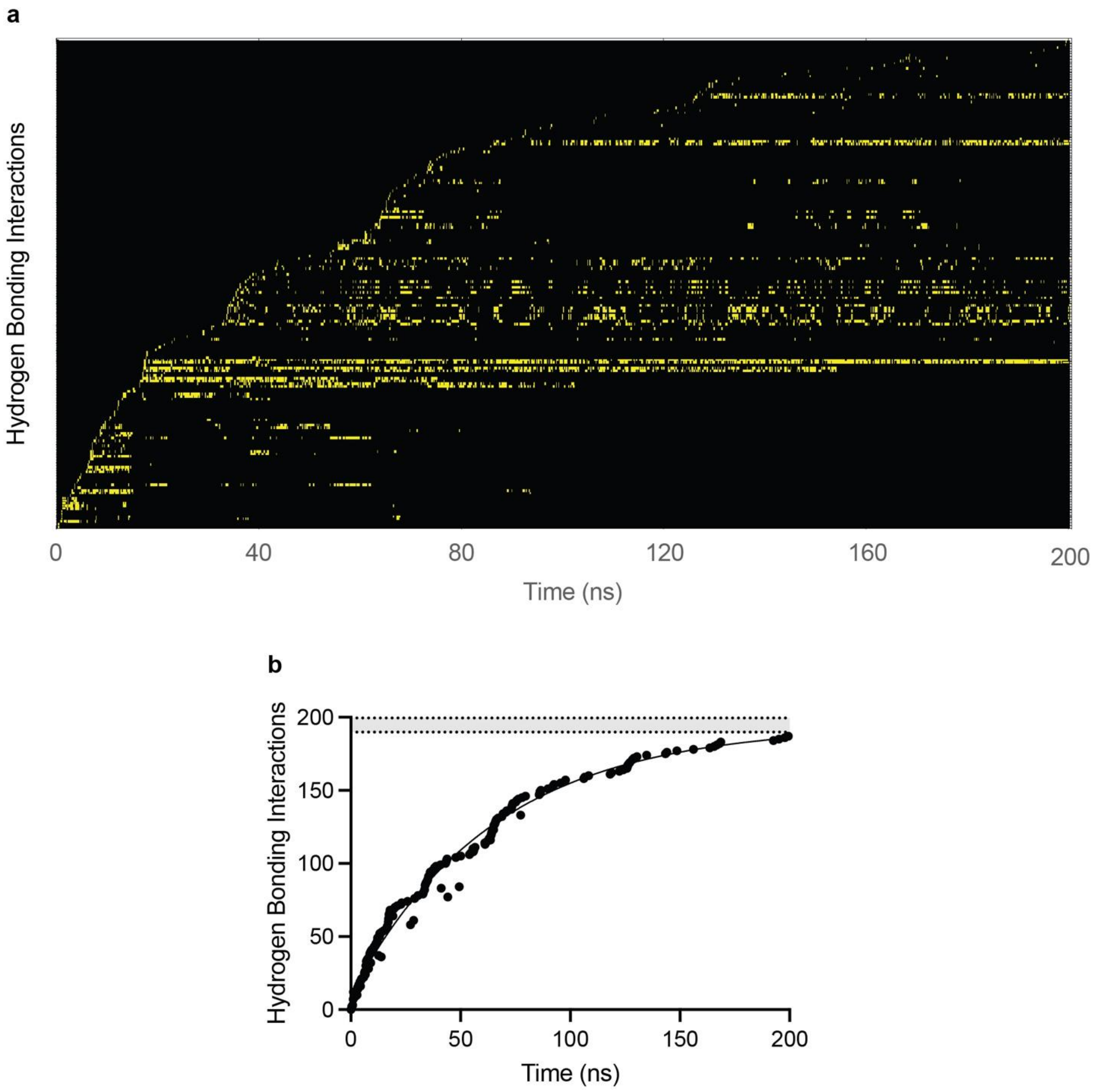

Supplementary Figure 9. **a.** A kymograph of all hydrogen bonding interactions during the course of the MD simulation. **b.** Non-linear fitting of the kymograph to an exponential plateau function. The shaded area indicates the asymptote of 195.4 with 95% CI [189.8,199.5].

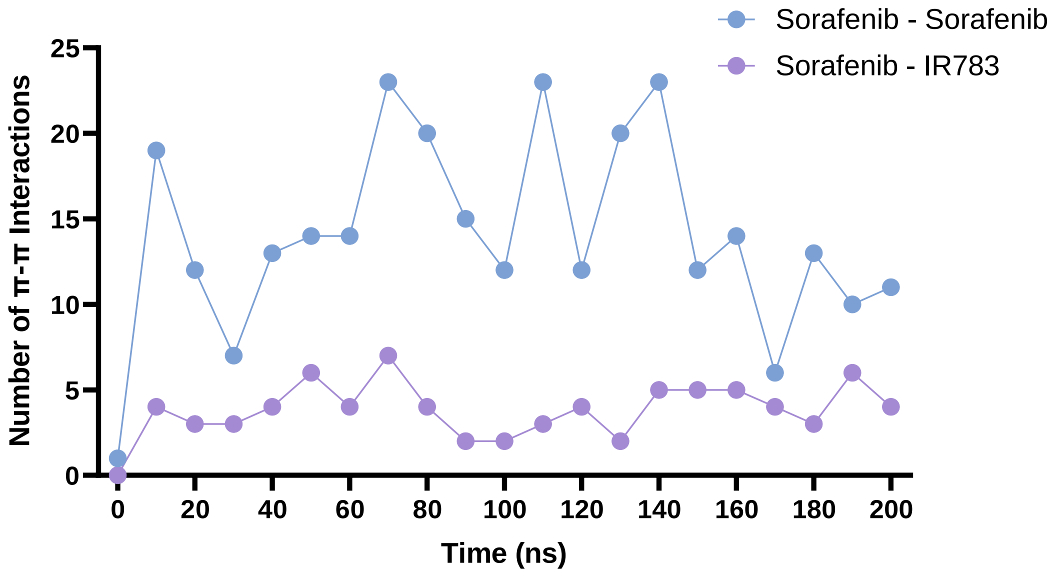

Supplementary Figure 10. Number of π-π intermolecular interactions between sorafenib molecules and between sorafenib and IR783 molecules during the course of the simulation. Source data are provided as a Source Data file.

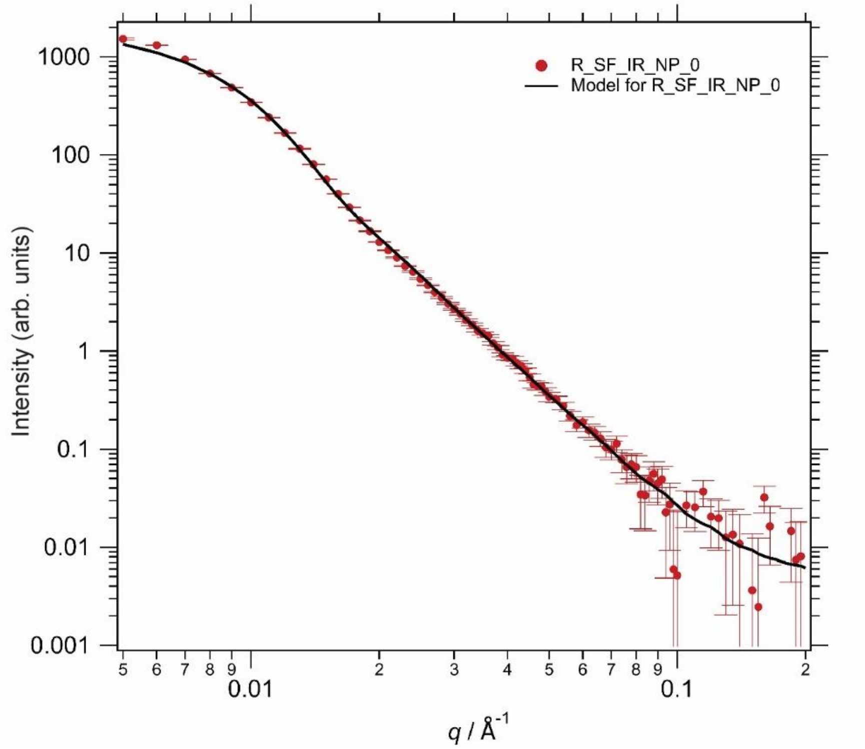

Supplementary Figure 11. Analysis of the solution scattering data of sorafenib nanoaggregates (Figure 4e). Using a spherical model for form factor, a radius of gyration (R_g) of 250 (±63.4) Å was obtained. Error bars = standard deviation.

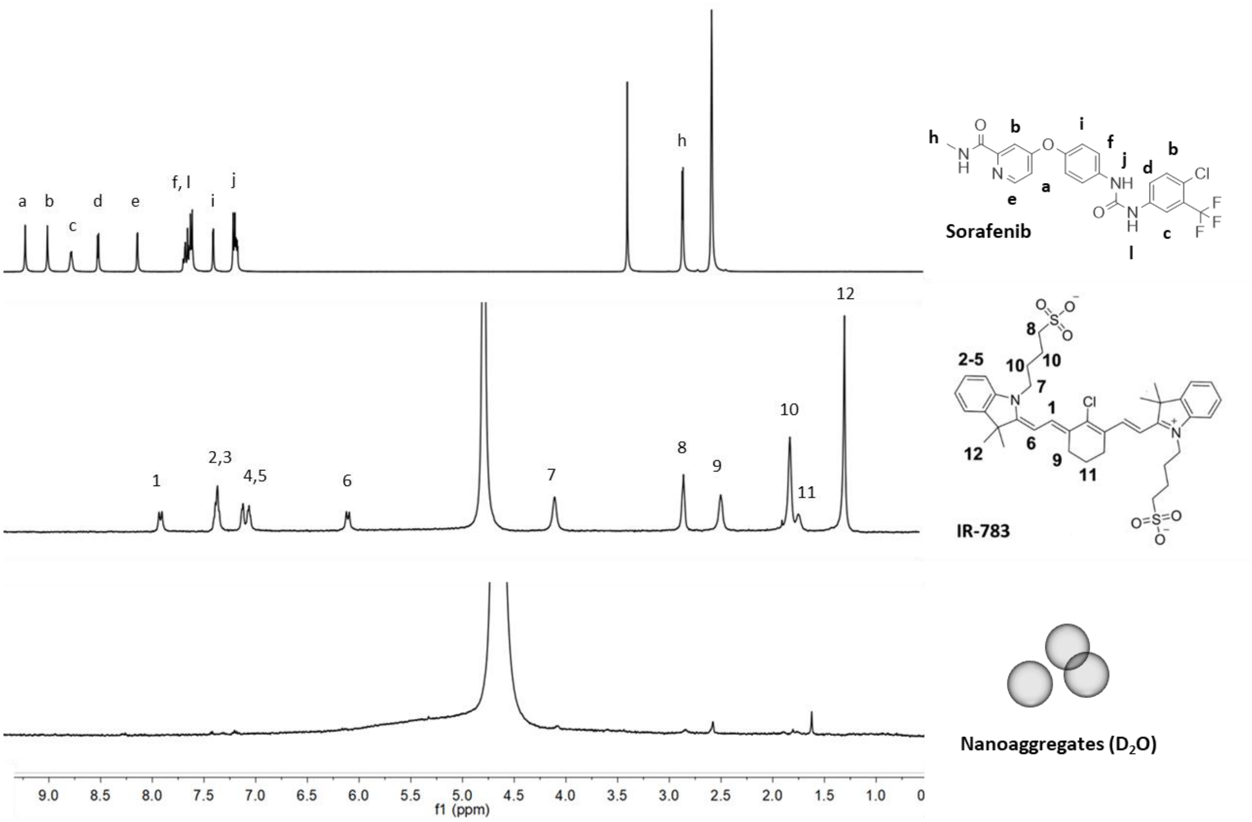

Supplementary Figure 12. NMR spectra for sorafenib, IR783 and sorafenib-IR783 nanoaggregates.

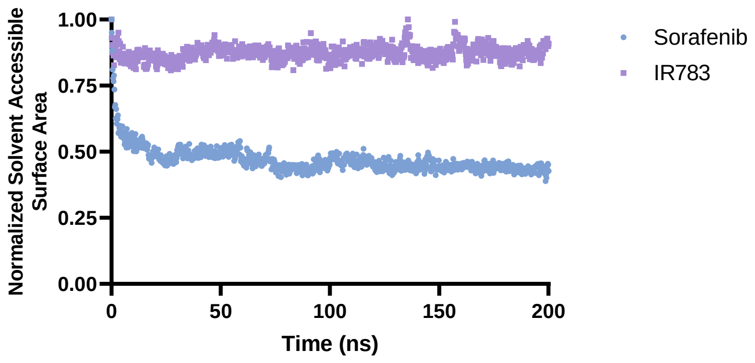

Supplementary Figure 13. Normalized solvent accessible surface area of both sorafenib and IR783 during the MD simulation normalized to time zero. Source data are provided as a Source Data file.

Supplementary Table 1. Fragment library for drug nanoaggregation assessments

Fragment/ Compound #	Structure	Name	Source	Size (nm)	PDI (a.u.)
1	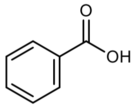	Benzoic acid	Sigma Aldrich	**N.A.***	**N.A.***
2	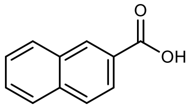	Naphthalene-2-carboxylic acid	Sigma Aldrich	1807.33±241.79 (IR783) 1510.33±60.01 (ICG)	0.2±0.04 (IR783) 0.28±0.03 (ICG)
3	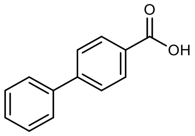	4-phenylbenzoic acid	Thermo Fisher Scientific	175.80±20.73 (IR783) 259.53±33.28 (ICG)	0.22±0.04 (IR783) 0.22±0.03 (ICG)
4	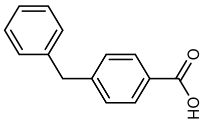	4-benzylbenzoic acid	Sigma Aldrich	229.57±27.81 (IR783) 232.10±50.22 (ICG)	0.28±0.03 (IR783) 0.31±0.03 (ICG)
5	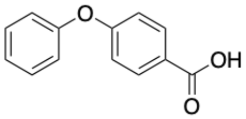	4-phenoxybenzoic acid	Thermo Fisher Scientific	462.30±18.71 (IR783) 340.07±19.39 (ICG)	0.23±0.02 (IR783) 0.21±0.03 (ICG)
6	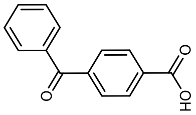	4-benzoylbenzoic acid	Sigma Aldrich	421.20±52.78 (IR783)	0.23±0.02 (IR783)
7	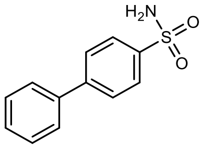	4-phenylbenzenesulfonamide	Enamine	221.83±2.94 (IR783) 187.93±9.99 (ICG)	0.27±0.03 (IR783) 0.22±0.01 (ICG)
8	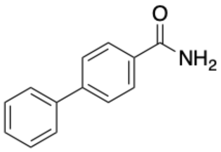	4-phenylbenzamide	Enamine	326.27±26.60 (IR783) 183.27±17.63 (ICG)	0.27±0.03 (IR783) 0.22±0.01 (ICG)

9	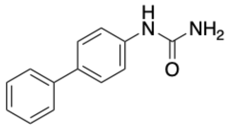	(4-phenylphneyl)urea	Enamine	311.33±28.91 (IR783) 289.20±10.45 (ICG)	0.28±0.01 (IR783) 0.18±0.01 (ICG)
10	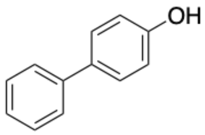	4-phenylphenol	Sigma Aldrich	711.00±43.66 (IR783) 483.60±15.31 (ICG)	0.30±0.06 (IR783) 0.29±0.02 (ICG)
11	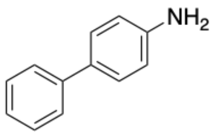	4-phenylaniline	Sigma Aldrich	**N.A.****	**N.A.****
12	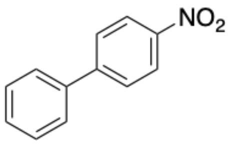	1-nitro-4-phenylbenzene	AA Blocks	1191.77±153.4 (IR783) 923.33±114.12 (ICG)	0.27±0.09 (IR783) 0.21±0.08 (ICG)
13	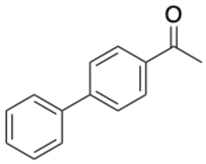	1-(4-phenylphenyl)-ethanone	Thermo Fisher Scientific	**N.A.***	**N.A.***
14	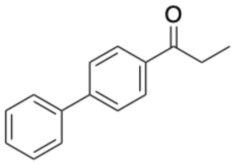	1-(4-phenylphenyl)propan-1-one	Sigma Aldrich	**N.A.****	**N.A.****
15	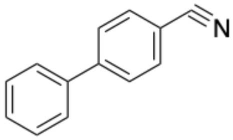	4-phenylbenzonitrile	Sigma Aldrich	**N.A.****	**N.A.****
16	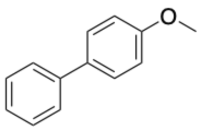	4-phenylphenol	Sigma Aldrich	1278.33±135.4 (IR783) 466.57±14.24 (ICG)	0.22±0.14 (IR783) 0.31±0.08 (ICG)

17	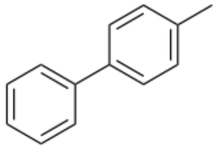	1-methyl-4-phenylbenzene	Sigma Aldrich	**N.A.****	**N.A.****
18	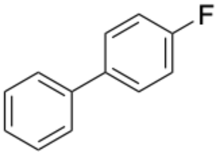	1-fluoro-4-phenylbenzene	Thermo Fisher Scientific	1095.00±34.65 (IR783) 574.83±6.48 (ICG)	0.42±0.04 (IR783) 0.24±0.03 (ICG)
19	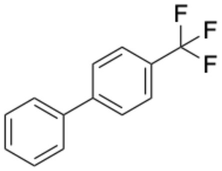	1-phenyl-4-(trifluoromethyl)benzene	Sigma Aldrich	1158.63±462.89 (IR783) 616.07±26.78 (ICG)	0.63±0.21 (IR783) 0.20±0.03 (ICG)
20	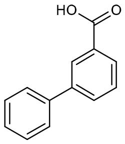	3-phenylbenzoic acid	Sigma Aldrich	634.7±198.68 (IR783) 1465.00±209.92 (ICG)	0.95±0.07 (IR783) 0.30±0.11 (ICG)
21	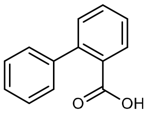	2-phenylbenzoic acid	Sigma Aldrich	**N.A.***	**N.A.***
22	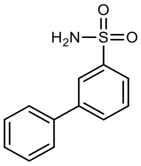	3-phenylbenzenesulfonamide	Enamine	**N.A.****	**N.A.****
23	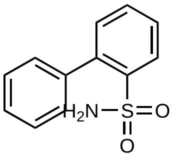	2-phenylbenzenesulfonamide	Sigma Aldrich	**N.A.***	**N.A.***
24	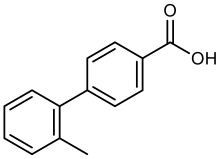	4-(2-methylphenyl)benzoic acid	Chembridge	136.92±58.95 (IR783) 145.75±53.84 (ICG)	0.17±0.03 (IR783) 0.18±0.02 (ICG)

25	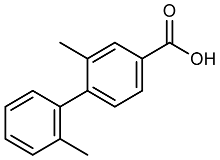	3-methyl-4-(2-methylphenyl)benzoic acid	AK Scientific	**N.A.****	**N.A.****
26E	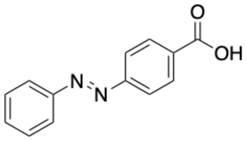	4-phenyldiazenylbenzoic acid (E)	Sigma Aldrich	130.20±10.05 (IR783) 92.38±34.77 (ICG)	0.14±0.01 (IR783) 0.2±0.01 (ICG)
26Z	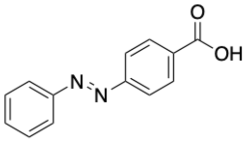	4-phenyldiazenylbenzoic acid (Z)	Sigma Aldrich	**N.A.***	**N.A.***
27	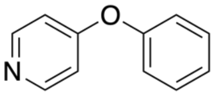	4-phenoxypyridine	Thermo Fisher Scientific	**N.A.***	**N.A.***
28	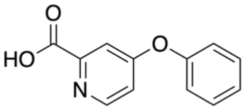	4-phenoxypyridine-2-carboxylic acid	Enamine	**N.A.***	**N.A.***
29	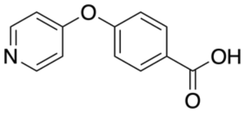	4-pyridin-4-yloxybenzoic acid	Wuxi AppTec	**N.A.***	**N.A.***
30	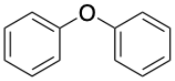	Phenoxybenzene	Sigma Aldrich	**N.A.****	**N.A.****
31	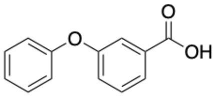	3-phenylbenzoic acid	Selleck Chemicals	1653.67±115.02 (IR783) 2041.67±233.64 (ICG)	0.48±0.05 (IR783) 0.23±0.03 (ICG)

32	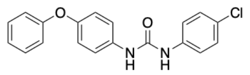	1-(4-chlorophenyl)-3-(4-phenoxyphenyl)urea	Synthesis (See Methods)	130.08±26.78 (IR783) 160.20±16.28 (ICG)	0.15±0.01 (IR783) 0.22±0.02 (ICG)
33	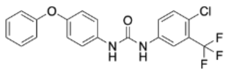	1-[4-chloro-3-(trifluoromethyl)phenyl]-3-(4-phenoxyphenyl)urea	Synthesis (See Methods)	81.11±6.42 (IR783) 118.73±5.35 (ICG)	0.11±0.02 (IR783) 0.24±0.01 (ICG)
34	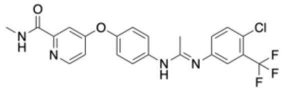	Sorafenib Analogue 1	Wuxi AppTec	**N.A.****	**N.A.****
35	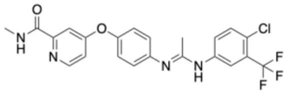	Sorafenib Analogue 2	Wuxi AppTec	**N.A.****	**N.A.****
36	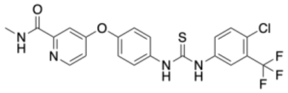	Sorafenib Analogue 3	Wuxi AppTec	90.74±29.43 (IR783) 113.91±11.92 (ICG)	0.32±0.01 (IR783) 0.21±0.02 (ICG)

*Not Applicable for dynamic light scattering (DLS) due to the samples were soluble with insufficient count rates in DLS.
** Not Applicable for DLS due to the samples formed large precipitations and could not be resuspended.

Supplementary Table 2. Solubility of selected fragments and drugs

Fragment or drug #	CAC in 7% DMSO (µg/mL)	CLogP	Intrinsic Solubility (µg/mL)
1	>1400	1.631	4305
2	79.99	2.620	70
3	30.73	3.278	23
7	11.43	2.227	40
20	30.53	3.278	23
21	>1400	3.278	23
22	272.68	2.227	40
23	>1400	2.227	40
24	20.42	3.791	8
25	17.3	4.305	2
Sorafenib	<5	3.183	0
Trametinib	5.22	3.183	1
Regorafenib	<5	4.486	0
